# FMNL1 Exhibits Pro-Metastatic Activity *via* CXCR2 in Clear Cell Renal Cell Carcinoma

**DOI:** 10.3389/fonc.2020.564614

**Published:** 2020-11-26

**Authors:** Mei-Fang Zhang, Qiu-Li Li, Yu-Feng Yang, Yun Cao, Chris Zhiyi Zhang

**Affiliations:** ^1^ Department of Pathology, Sun Yat-sen University Cancer Center, State Key Laboratory of Oncology in South China, Collaborative Innovation Center for Cancer Medicine, Guangzhou, China; ^2^ Department of Head and Neck Surgery, Sun Yat-sen University Cancer Center, State Key Laboratory of Oncology in South China, Collaborative Innovation Center for Cancer Medicine, Guangzhou, China; ^3^ Department of Pathology, Dongguan Third People’s Hospital, Dongguan, China; ^4^ Key Laboratory of Functional Protein Research of Guangdong Higher Education Institutes and MOE Key Laboratory of Tumor Molecular Biology, Institute of Life and Health Engineering, College of Life Science and Technology, Jinan University, Guangzhou, China

**Keywords:** FMNL1, GATA3, CXCR2, tumor metastasis, clear cell renal carcinoma

## Abstract

Formin-like (FMNL) proteins are responsible for cytoskeletal remodeling and have been implicated in the progression and spread of human cancers. Yet the clinical significance and biological function of FMNL1 in clear cell renal cell carcinoma (ccRCC) remain unclear. In this study, the expression of FMNL1 in ccRCC and its clinical value were determined by tissue microarray-based IHC and statistical analyses. The role of FMNL1 in ccRCC metastasis and the underlying mechanism were investigated *via in vitro* and *in vivo* models using gene regulation detection, ChIP, Luciferase reporter assays, and rescue experiments. We show that FMNL1 is upregulated in ccRCC and exhibits pro-metastatic activity *via* induction of CXCR2. High expression of FMNL1 is significantly correlated with advanced tumor stage, higher pathological tumor grade, tumor metastasis, and unfavorable prognosis in two independent cohorts containing over 800 patients with ccRCC. The upregulation of FMNL1 in ccRCC is mediated by the loss of GATA3. Ectopic expression of FMNL1 promotes, whereas FMNL1 depletion inhibits cell migration *in vitro* and tumor metastasis *in vivo*. The FMNL1-enhanced cell mobility is markedly attenuated by the knockdown of CXCR2. Further studies demonstrate that FMNL1 increases the expression of CXCR2 *via* HDAC1. In clinical samples, FMNL1 expression is positively associated with CXCR2, and is negatively connected to GATA3 expression. Collectively, our data suggest FMNL1 serve as a potential prognostic factor and function as an oncogene. The axis of GATA3/FMNL1/CXCR2 may present a promising therapeutic target for tumor metastasis in ccRCC.

## Introduction

Clear cell renal cell carcinoma (ccRCC) represents one of the most common malignancies in the urinary system, accounting for 2% of total cancer patients (1). In 2018, more than 400 thousand new ccRCC cases were diagnosed. The patients’ 5-year survival rate could be up to 92% if tumors were restricted in the kidney, but dramatically drops down to12-67% if tumors metastasized to other organs (2). Unfortunately, about 30% of ccRCC patients were initially diagnosed with tumor metastasis, and 20–40% of the patients receiving surgical resection experienced tumor relapse with lymphatic or distal tumor metastasis (3). Current therapeutic strategies targeting metastatic ccRCC failed to improve the prognosis of cancer patients. As a result, it is of clinical significance to disclose the mechanism of ccRCC metastasis.

Cell motility is attributed to the polymerization of filamentous actin networks regulated by formins, a highly conserved family of cytoskeletal remodeling proteins. Formin-like protein 1 (FMNL1) helps to organize actin filaments into bundles to modulate the actin dynamics, participating in Golgi complex stability, phagocytosis and podosome dynamics (4). Literatures reported that FMNL1 was naturally expressed in hematopoietic cells and immune organs including thymus and spleen, but not in liver and kidney (5). A growing body of evidence showed that FMNL1 was overexpressed in acute lymphoblastic, myeloid, promyelocytic leukemia, and chronic myelogenous leukemia to promote the pathogenic progression (6–8). Strikingly, FMNL1 was also found to be upregulated in solid tumors, such as nasopharyngeal carcinoma (9), non-small cell lung cancer (10), and gioblastoma (11). Functionally, FMNL1 was able to activate the epithelial to mesenchymal transition (EMT) to promote tumor progression (9, 10). However, the role FMNL1 in ccRCC remains unclear.

Using tissue microarray (TMA)-based immunohistochemistry, *in vitro* and *in vivo* experiments, we show that FMNL1 expression in ccRCC is increased and associated with poor outcomes. High expression of FMNL1, with the help of HDAC1, upregulates CXCR2 to trigger EMT process and to facilitate cell migration and tumor metastasis in ccRCC. Our data suggest FMNL1 serve as a pro-metastatic oncogene in ccRCC.

## Materials and Methods

### Patients and Specimens

A cohort containing 306 paraffin-embedded ccRCC and the corresponding adjacent nontumorous tissues were obtained for immunohistochemistry (IHC) studies from Sun Yat-sen University Cancer Center (SYSUCC), Guangzhou, China with prospective collection of clinicopathological information from March 2010 to September 2012. Another 20 pairs of ccRCC fresh specimens were also collected for the determination of FMNL1 mRNA and protein expression. All patients did not receive chemotherapy or radiotherapy before surgery. Written informed consent was obtained from each patient. This project was approved by the Sun Yat-sen University Cancer Center Institute Research Ethics Committee. The expression and clinical significances or FMNL1 in ccRCC were further confirmed in studies from Gene Expression Omnibus (GEO) (https://www.ncbi.nlm.nih.gov/sites/GDSbrowser?acc=GDS505), Oncomine (https://www.oncomine.org/), The Cancer Genome Atlas (TCGA) (http://www.cbioportal.org/) and Clinical Proteomic Tumor Analysis Consortium (CPTAC) (https://cptac-data-portal.georgetown.edu/) datasets.

### Cell Culture and Transfection

ccRCC cell lines (ACHN, RCC4, RCC10, A498, and 786-O) cells were obtained from the Cell Resource Center, Chinese Academy of Science Committee (Shanghai, China), and maintained in Dulbecco’s modified Eagle’s medium (DMEM) (Gibco, Gaithersburg, MD, USA) supplemented with 10% heat-inactivated fetal bovine serum (FBS, Hyclone, Logan, UT) in a humidified incubator at 37°C and 5% CO_2_. Overexpression vector encoding full-length human FMNL1 cDNA was transfected into A498 and RCC10 cells, using Lipofectamine 2000 (Invitrogen) according to the manufacturer’s instructions. Cells transfected with an empty pcDNA 3.1+ vector were used as controls. Cells were selected by G418 for 2 weeks to establish stable cell lines. The 786-O cells were infected with retroviruses carrying psi-LVRH1MP-FMNL1-shRNAs, according to the following sequence: shRNA-1: CATCGCGCCATCATGAACTAC; shRNA-2: GGAGAYGAAGTCGACTGACG. Stable cell lines were applied in the *in vitro* and *in vivo* investigations of the biological function of FMNL1 in ccRCC.

### Quantitative Real-Time Polymerase Chain Reaction (qRT-PCR)

mRNA transcripts were measured using a standard SYBR Green real-time assay. Total RNA was extracted from cells using the Trizol reagent (Invitrogen, CA, USA) according to the manufacturer’s instruction. One microgram of RNA sample was reverse transcribed using the Superscript III enzyme (Invitrogen, CA, USA) to obtain single-stranded cDNA. Real-time PCR was then performed on cDNA in an iQSybr Green Supermix (Bio-Rad) with gene-specific primers. The following primers were used: FMNL1, forward: 5’- GAAGCTGAAGAGCTATGTGG-3’ and reverse: 5’- CCTGCGTGGACTCCTGAACT-3’; β-actin, forward: 5′-TGGCACCCAGCACAATGAA-3′ and reverse: 5′-CTAAGTCATAGTCCGCCTAGAAGCA-3′. Amplicons were analyzed using the -ΔCt method.

### Western Blot

Total proteins were extracted and separated by 10% SEMS-PAGE and then transferred onto PVDF membrane (Millipore, Bedford, MA). Equal amounts of protein (30 μg) were resolved by SDS-PAGE and then electrophoretically transferred onto PVDF membranes. After blocked in 5% non-fat milk 1 h at room temperature, the membranes were incubated with appropriately diluted primary antibodies overnight at 4°C. After washed thrice with TBST, The blotted membranes were incubated with primary antibodies for FMNL1 (1:200, LS-B8887, LifeSpan BioSciences, Seattle, WA), HDAC1 (1:500, sc-81598, Santa Cruz Biotechnology, Santa Cruz, CA), CXCR2 (1:200, ab14935, Abcam, Cambridge, MA), E-cadherin (1:1,000, #14472, Cell Signaling Technology), β-catenin (1:1,000, #9562, Cell Signaling Technology), N-cadherin (1:1,000, #13116, Cell Signaling Technology), and Vimentin (1:1,000, #5741, Cell Signaling Technology). The membranes were incubated with HRP-conjugated secondary antibody at 1:20,000 dilutions for 1 h at room temperature. The membranes were visualized by the enhanced Phototope TM-HRP Detection Kit and exposed to Kodak medical X-ray processor (Carestream Health, USA). Anti-β-actin (1:1,000, #4970, Cell Signaling Technology) was used as a loading control.

### Immunohistochemistry and Evaluation

For immunohistochemical studies, paraffin embedded sections were dewaxed in xylene (3 × 5 min) and dehydrated in ethanol series (3 min in 100% ethanol, 1 min in each of 95 and 70% ethanol). Sections were washed in PBS and endogenous peroxidases were blocked with 3% H2O2 for 10 min. The tissue sections were subjected to antigen retrieval by pressured cooking in 10 mM citrate buffer for 3 min, and then incubated with serum blocking solution for 20 min to block nonspecific binding, followed by incubation with primary antibodies for 2 hours at room temperature. After rinsing in PBS for 10 min, the sections were incubated with the biotinylated secondary antibody for 1 h and further incubation with the Streptavidin Biotin complex. Reactivity was developed in chromogen DAB (3,3-diaminobenzidine) solution. The signal was enhanced by applying the solution of CuSO_4_ and NaCl for 5 min. Finally, the sections were counterstained with Mayer’s hematoxylin, dehydrated, and mounted. All sections were observed under light microscopy and the staining intensities were assessed by two independent pathologists (Yang YF and Cao Y). Nucleus staining was graded for intensity (0-negative, 1-weak, 2-moderate, and 3-strong) and percentage of positive cells [0, 1 (1–24%), 2 (25–49%), 3 (50–74%), and 4 (75–100%)] with discrepancies resolved by consensus. The H-scores for tumors with multiple cores were averaged. The median IHC score was used to define high FMNL1 expression and low FMNL1 expression groups.

### Transwell Assay

A total of 3 × 104 cells were re-suspended in 200 μl of serum-free medium and placed in the upper compartment of a Transwell chamber (Corning; 24-well insert, pore size: 8 μm) with (Invasion assay) or without (Migration assay) extracellular matrix gel (BD Biosciences, San Jose, CA, USA). The lower chamber was filled with 15% fetal bovine serum as a chemoattractant and incubated for 48 h for the migration assay. The cells on the upper surface of the membrane were removed, and the cells on the lower surface were fixed and stained with 0.05% crystal violet. Five visual fields of each insert were randomly chosen and counted under a light microscope.

### Animal Model

Approximately 5 × 10^5^ stable cells were injected *via* the tail vein BALB/c-nude mice (4-weeks age, six mice per group). After 6 weeks, mice were sacrificed. The lungs were fixed in 4% paraformaldehyde, serially sectioned and stained with hematoxylin and eosin (HE). Lung metastasis was counted and quantified in serial selections of high-power fields. The tumor number was obtained through the counting of focal tumors in serial sections of the lungs. All animal studies were approved by the Medical Experimental Animal Care Commission of Sun Yat-sen University Cancer Center.

### Chromatin Immunoprecipitation Assay

Chromatin Immunoprecipitation Assay (ChIP) was performed according to the manufactory instruction of ChIP kit (Millipore, 17-10,085 and 17-10,086). Briefly, RCC10 cells transfected with GATA3 overexpression vector were cultured in 100 mm plates to grow to 70–80% confluence. Cells were fixed by fixative solution (1/10th the volume of growth medium volume), and then treated with stop solution (1/20th the volume of growth medium volume). Cells were centrifugated and resuspended in ChIP buffer to collect nuclear pellet. The cell lysate was then sonicated and incubated with 5 mg of GATA3 or H3K9ac antibody at 4°C overnight. After incubating with protein A/G agarose beads for 4 h at 4°C, the bound DNA-protein complexes were eluted and washed to reverse the cross-links. DNAs were extracted and purified for PCR detection. The primers for the promoters are as following: FMNL1 forward: 5’- CCGCTGCTTTGTCTTTGTCC-3’; reverse: 5’- CGCAGGGACCCCTGATATTC-3’. CXCR2 forward: 5’-GCATACAGTTTCAGGGAAAGAG-3’; reverse: 5’-CGCTAGCTATTGATGCAGTCT-3’. ECHS1 forward: 5’-CTGGTCTCAAACTCCTGACGT-3’; reverse primer 5’-CCATTTGTGTACTTGCCCGGAT-3’. ECHS1 was used as a positive control for GATA3-mediated ChIP assays (12).

### Luciferase Reporter Assay

RCC10 cells were co-transfected with GATA3 overexpression vector and 500 ng of psiCHECK-2-FMNL1-promoter. Cells were collected 36 h after transfection and analyzed with the Dual-Luciferase Reporter Assay System (Promega, CA, USA).

### Gene Set Enrichment Analysis

Gene set enrichment analysis (GSEA) was conducted using the java (GSEA 3.0)–based graphical user interface using a pre-ranked list with the classic enrichment statistic. GSEA involves determining whether a predefined set of genes is significantly different between the two groups: high and low FMNL1. The entire list of genes is ranked according to expression difference of FMNL1 in HCC samples form TCGA database. An enrichment score for each gene set is then calculated. Cumulative distribution function was constructed by performing 1,000 random gene set membership assignments.

### Statistical Analysis

Data from three separate experiments are presented as mean ± SED. The Student’s t-test was used for comparisons between groups unless otherwise noted. Kaplan–Meier analyses were used for survival analysis. Differences were considered significant for *P*-values less than 0.05.

## Results

### FMNL1 Expression Is Increased in ccRCC and Correlated With Poor Outcomes

**Figure 1:**
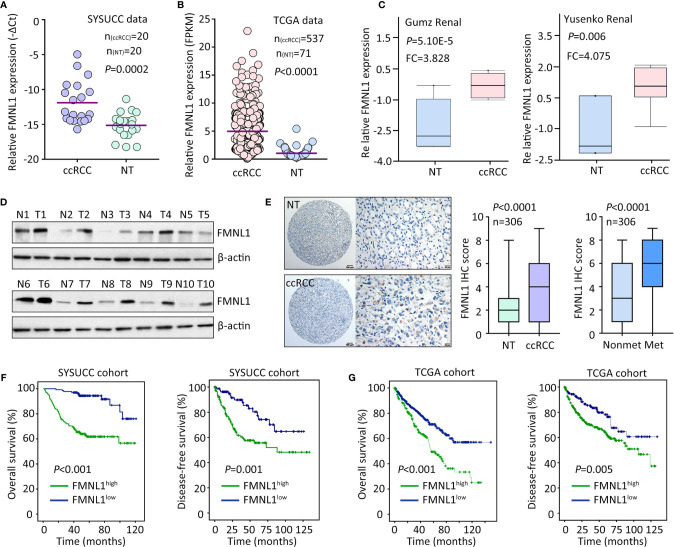
FMNL1 is overexpressed in ccRCC. **(A)** Total RNA was extracted from 20 paired ccRCC and corresponding nontumorous tissues (NT) to examine the expression of FMNL1 mRNA by qRT-PCR. **(B)** The mRNA level in ccRCC patients from TCGA dataset was shown **(C)**. The upregulation of FMNL1 mRNA expression in ccRCC tissues was validated in previous studies of Gumz and Yusenko. **(D)** The protein expression of FMNL1 was examined by western blot in 20 ccRCC specimens. Representative images were shown. **(E)** A cohort containing 306 ccRCC patents was recruited to construct TMA. IHC was performed to indicate the FMNL1 expression. FMNL1 expression in tumor and nontumorous tissues, and in ccRCC tissues with or without tumor metastasis was indicated by box and whiskers. **(F)** Patients were divided into FMNL1^high^ and FMNL1^low^ groups, according to the IHC score of FMNL1 in ccRCC tissues. Kaplan-Meier survival analyses were performed to reveal the prognostic value of FMNL1 in overall and disease-free survivals of ccRCC patients. **(G)** The prognostic value of FMNL1 in ccRCC was confirmed in TCGA cohort.

The mRNA and protein expression of FMNL1 was determined in ccRCC samples. qRT-PCR showed that FMNL1 mRNA level in ccRCC specimens was significantly higher than that in the nontumorous tissues adjacent to tumor (**Figure 1A**). The upregulation of FMNL1 mRNA in ccRCC tissues was confirmed in studies from TCGA, GSD505, Gumz renal and Yusenko renal (**Figures 1B, C** and **Supplementary Figure 1**). The increase of FMNL1 mRNA was most obvious among TCGA cancers, although differential expression of FMNL1 was recorded (**Supplementary Figure 2**). Consistently, the protein expression of FMNL1 in ccRCC tissues was markedly increased, compared to the nontumorous tissues (**Figure 1D**). CPTAC data also presented higher expression of FMNL1 protein in ccRCC tissues (**Supplementary Figure 3**). In a cohort containing 306 ccRCC cases, FMNL1 protein detected by IHC expressed in the cytoplasm of cancer cells (**Figure 1E**). Higher expression of FMNL1 (ccRCC vs. NT) was depicted in 77.8% (238/306) of the cases, whereas lower expression was found in 16.7% (51/306) of the cases. Strikingly, FMNL1 expression in patients with tumor metastasis was much higher than that in patients without tumor metastasis (**Figure 1E**). This may imply that FMNL1 is involved in the ccRCC progression.

The clinical value of FMNL1 in ccRCC was next determined. In TCGA cohort, high FMNL1 expression was associated with tumor pathological grade, clinical stage and tumor metastasis (**Supplementary Figure 4**). Using the IHC score (5.23) determined by ROC curve, patients were separated into two groups: FMNL1 high and FMNL1 low. About 56.2% (172/306) of patients were with high expression of FMNL1. High expression of FMNL1 was significantly associated with Fuhrman score and lymph node invasion in SYSUCC cohort (**Supplementary Table 1**). Prognostic analyses indicated that high expression of FMNL1 was significantly correlated with overall and disease-free survivals in both SYSUCC and TCGA cohort (**Figures 1F, G**). Patients expressing more FMNL1 in tumor tissues survival much shorter and experienced tumor relapse in a much shorter time. Collectively, these findings indicate that FMNL1 overexpression in ccRCC may serve as a potential factor.

### FMNL1 Upregulation Is Mediated by the Loss of GATA3 in ccRCC

**Figure 2:**
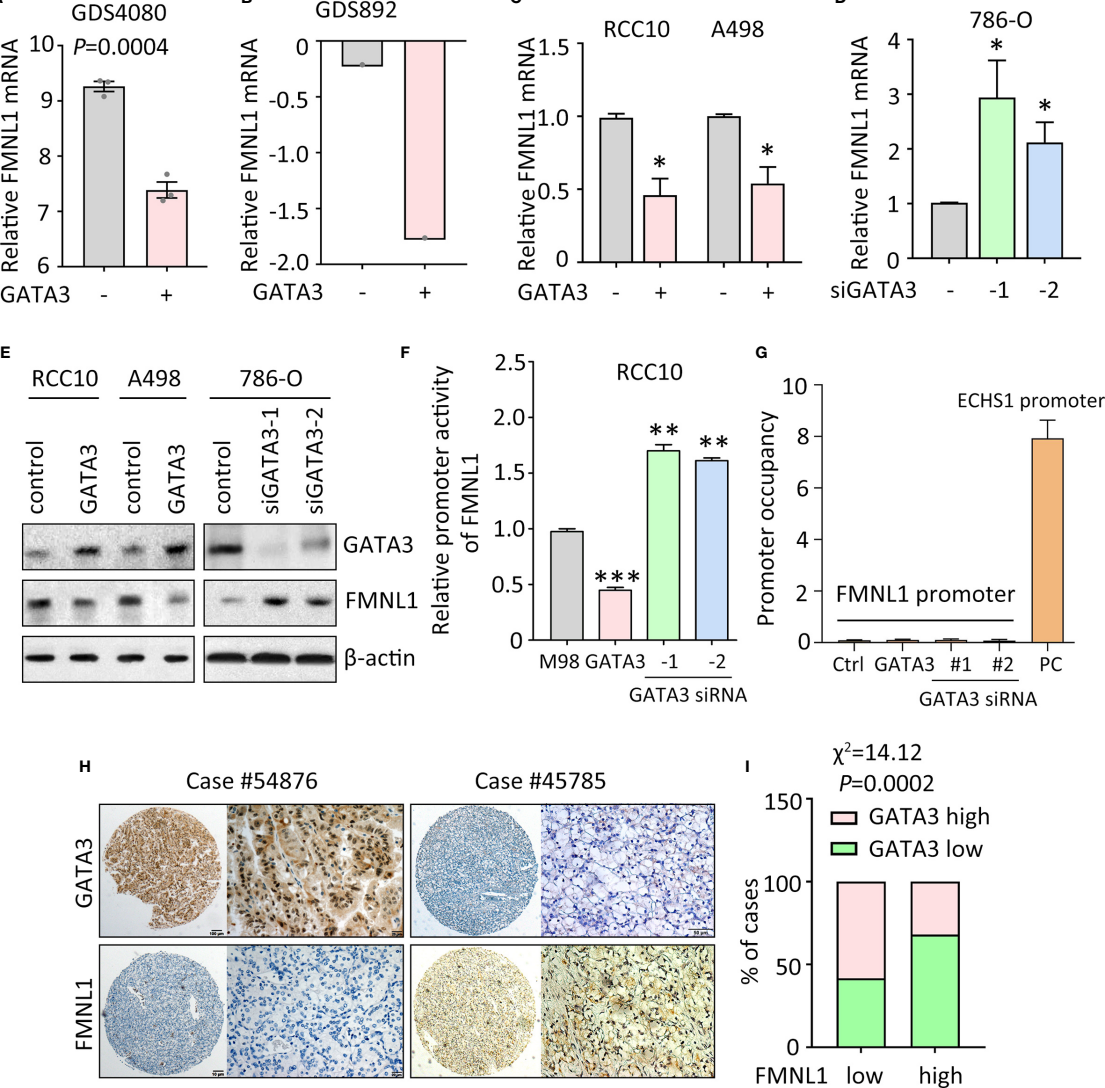
FMNL1 is regulated by GATA3 in ccRCC cells. **(A)** The expression of FMNL1 was determined in breast cancer MDA-MB-231 cells. Data were from a GEO study (no. GDS4080). **(B)** 293T cells were transfected with GATA3. The mRNA expression of FMNL1 was examined. Data were from another GEO study (no. GDS892). **(C)** RCC10 and A498 cells were transfected with GATA3 overexpression vectors for 36 h. The expression of FMNL1 mRNA was examined by qRT-PCR. *P < 0.05. **(D)** FMNL1 mRNA levels in 786-O cells transfected with GATA3 siRNAs for 36 h were measured. *P < 0.05. **(E)** The protein expression of GATA3 and FMNL1 in ccRCC cells with GATA3 overexpression or knockdown was examined by western blot. **(F)** Dual luciferase reporter assays were performed to indicate the effect of GATA3 on the activity of FMNL1 promoter. **P < 0.01, ***P < 0.001. **(G)** ChIP assays were used to detect the enrichment of GATA3 on FMNL1 promoter. The binding of GATA3 onto ECHS1 promoter was used as positive control (PC). **(H)** GATA3 expression was further examined in 306 ccRCC paraffin-embedded tissues. A ccRCC case with low expression of FMNL1 showed high GATA3 expression. **(I)** The correlation of GATA3 and FMNL1 expression was determined by Pearson correlation analyses in 306 ccRCC cases.

Data of our study and TCGA indicated that FMNL1 was upregulated in most of the clinical samples. According to the TCGA data, gene amplification and promoter demethylation were insufficient to support the FMNL1 upregulation (**Supplementary Figure 5** and data not shown). Bioinformatics analyses indicated that binding sites for GATA transcription factors were available in the promoter of FMNL1 (data not shown). Previous studies showed that GATA3 was capable of suppressing ccRCC metastasis (13). Interestingly, in GEO studies (no. GDS4080 and GDS892), overexpression of GATA3 inhibited the mRNA expression of FMNL1 (**Figures 2A, B**). Ectopic expression of GATA3 in RCC10 and A498 cells decreased, whereas knockdown of GATA3 by siRNAs in786-O cells increased the mRNA and protein expression of FMNL1 (**Figures 2C–E**). To further demonstrate the regulation of FMNL1 by GATA3, dual luciferase reporter and ChIP assays were performed. Results showed that overexpression of GATA3 markedly inhibited the activity of FMNL1 promoter (**Figure 2F**). However, ChIP assays demonstrated that GATA3 was not able to bind to FMNL1 promoter (**Figure 2G**), suggesting that GATA3 indirectly regulate the expression of FMNL1. Reverse correlation between GATA3 and FMNL1 was confirmed in TCGA and our samples. The GATA3 mRNA and protein expression in TCGA ccRCC tissues was much lower than that in nontumorous tissues (**Supplementary Figures 6 and 7**). In SYSUCC cohort, cases with high expression of GATA3 were frequently accompanied with low expression of FMNL1 (**Figures 2H, I**). These data indicate the expression of FMNL1 expression could be modulated by GATA3 in ccRCC.

### FMNL1 Exerts Pro-Metastatic Activity in ccRCC

**Figure 3:**
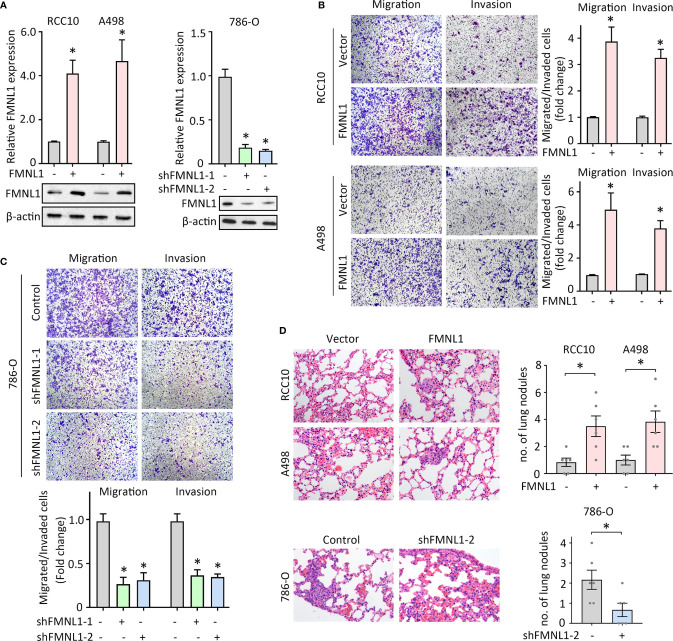
FMNL1 promotes ccRCC cell migration *in vitro* and tumor metastasis *in vivo*. **(A)** RCC10 and A498 cells were transfected with FMNL1 overexpression vector and selected by G418 for 2 weeks. The mRNA and protein expressions of FMNL1 in stable cells were examined by qRT-PCR and western blot. Stable 786-O cells with FMNL1 knockdown was also established. *P < 0.05. **(B**, **C)** Stable cells were seeded onto the transwell chamber with or without Matrigel coating and cultured for 36 h. The migrated or invaded cells were stained by 0.05% of crystal violet, and counted under a microscope the indicate the effect of FMNL1 overexpression **(B)** or silencing **(C)** on ccRCC cell migration. *P < 0.05. **(D)** Stable cells were injected into BALB/c nude mice through tail vein. After 40 days, lungs were dissected. HE staining were performed to show the metastatic nodules in the lungs. The number of tumors in lungs was shown by histogram. *P < 0.05.

FMNL1 expression was closely associated with tumor metastasis in our and TCGA cohort. Previous studies demonstrated that FMNL1 was responsible for the tumor progression in nasopharyngeal carcinoma and non-small cell lung cancer. The role of FMNL1 in ccRCC cell migration was next determined. Lentivirus-mediated overexpression and silencing of FMNL1 were applied in ccRCC cells. The alternation of mRNA and protein expression of FMNL1 was confirmed by qRT-PCR and western blot (**Figures 3A, B**). Migration and invasion assays were performed to evaluate the effect of FMNL1 on cell motility. Results showed that FMNL1 overexpression remarkably enhanced the cell migration and invasion (**Figure 3B**). On the other hand, FMNL1 depletion strongly weakened the capability of cell movement (**Figure 3C**). The tail vein injection metastasis nude mouse model was used to test the FMNL1-mediated tumor metastasis. Cells with FMNL1 overexpression formed more metastatic lung nodules, compared with the control groups. In contrast, FMNL1 knockdown substantially inhibited the tumor formation in the lung (**Figure 3D**). These findings demonstrate the pro-metastatic role of FMNL1 in ccRCC.

### FMNL1 Enhances Ccrcc Cell Migration *via* CXCR2

**Figure 4:**
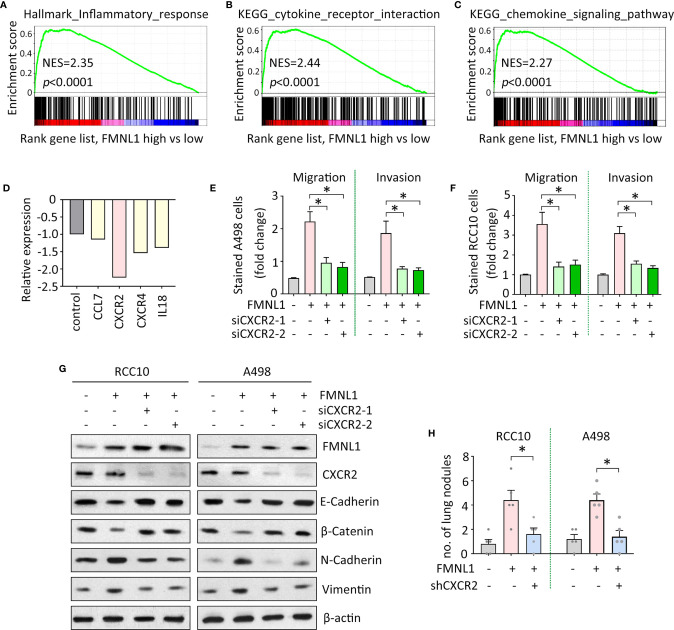
FMNL1 exerts pro-metastatic activity *via* CXCR2 in ccRCC. **(A**–**C)** Gene sets enrichment analysis (GSEA) based on TCGA data showed that pathways involved in inflammation were activated in ccRCC patients with high expression of FMNL1. **(D)** PCR microarrays containing 84 genes were used to disclose the mechanism of FMNL1-induced cell migration. Genes related to inflammation were chosen for further validation. **(E**, **F)** Cells with FMNL1 overexpression were transfected with CXCR2 siRNAs. Cell migration and invasion were determined by transwell assays in A498 **(E)** and RCC10 **(F)** cells *P < 0.05. **(G)** Proteins were extracted from cells treated as described in E were subjected into western blot to determine the expression of EMT markers, such as E-Cadherin, β-catenin, N-Cadherin and Vimentin. β-actin served as loading control. **(H)** CXCR2 shRNA was introduced into RCC10 and A498 stable cells with FMNL1 overexpression. Stable cells were injected into nude mice for 40 days. Metastatic nodules in the lungs were counted and indicated by histogram. *P < 0.05.

The mechanism of FMNL1-mediated tumor metastasis was next investigated. Gene set enrichment analysis (GSEA) based on the TCGA data indicated that genes involved in inflammation were enriched in patients with high expression of FMNL1 (**Figure 4A**). Signaling pathways of cytokine and chemokine were activated by FMNL1 (**Figures 4B, C**). Furthermore, Human Tumor Metastasis RT² Profiler™ PCR Array consisting of 84 well-known metastasis-related genes was used identify the potential downstream effectors of FMNL1 in ccRCC cells. In A498 cells with FMNL1 depletion, CXCR2 was noticeably downregulated, compared with other factors involved in inflammation (**Figure 4D**). The effect of CXCR2 on FMNL1-mediated malignant phenotype was examined. Transwell assays showed that knockdown of CXCR2 by siRNAs in A498 and RCC10 cells substantially attenuated the cell migration and invasion (**Figures 4E, F**). Epithelial-Mesenchymal Transition (EMT) has been demonstrated to be essential for tumor metastasis. The ectopic expression of FMNL1 in ccRCC cells triggered EMT to promote cell migration. Epithelial markers E-Cadherin and β-Catenin were downregulated, whereas mesenchymal markers N-Cadherin and Vimentin were upregulated by the overexpression of FMNL1. However, this was rescued by the knockdown of CXCR2 (**Figure 4G**). Data from *in vivo* model showed that knockdown of CXCR2 in stable cells with FMNL1 overexpression significantly reduced the metastatic nodules in the lungs (**Figure 4H**). These data suggest the pro-metastatic role of FMNL1 in ccRCC is partly supported by CXCR2.

### FMNL1 Induces CXCR2 Expression *via* HDAC1 in ccRCC cells

**Figure 5:**
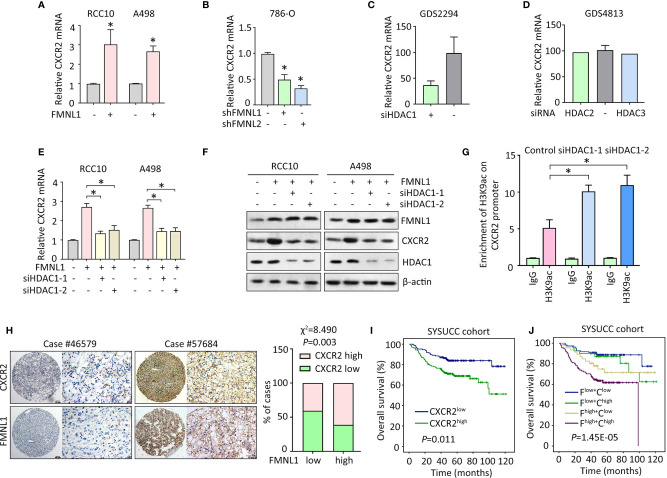
FMNL1 upregulates CXCR2 *via* HDAC1 in ccRCC cells. **(A**, **B)** The expression of CXCR2 mRNA in stable cells with FMNL1 overexpression **(A)** and knockdown **(B)** was determined by qRT-PCR. *P < 0.05. **(C**, **D)** CXCR2 mRNA expression was determined in cells treated with siRNA for HDAC1, HDAC2 or HDAC3 in human cancer cells. Data were from GEO datasets (no. GDS2294 and GDS4813). **(E**, **F)** Cells with FMNL1 overexpression were transfected with HDAC1 siRNAs for 36 h. CXCR2 mRNA **(E)** and protein **(F)** expressions were determined by qRT-PCR and western blot. *P < 0.05. **(G)** The histone H3 acetylation at lysine 9 was determined by ChIP assays in RCC10 cells with transfection of HDAC1 siRNAs. **(H)** CXCR2 expression was further examined in 306 ccRCC paraffin-embedded tissues. Representative IHC images showed positive correlation of CXCR2 and FMNL1 expression. The correlation of CXCR2 and FMNL1 expression was determined by *Pearson* correlation analyses in 306 ccRCC cases. **(I**, **J)** The effect of CXCR2 expression **(I)**, as well as its combination with FMNL1 expression **(J)**, on the overall survival time of ccRCC patients in SYSUCC cohort was determined. F, FMNL1; C, CXCR2.

Since CXCR2 was required for the oncogenic function of FMNL1 in ccRCC, the potential connection between the two molecules was determined. In RCC10 and A498 stable cells with FMNL1 overexpression, the mRNA level of CXCR2 was markedly increased (**Figure 5A**). Consistently, FMNL1 depletion resulted in decrease of CXCR2 mRNA (**Figure 5B**). This might implicit that FMNL1 was able to affect the transcription of CXCR2 in ccRCC cells. Current literatures showed that FMNL1 incorporates with HDAC1 to facilitate tumor metastasis and that CXCR2 expression could be modulated by HDAC proteins. Strikingly, according to the findings of GEO studies (no. GDS2294 and GDS4813), CXCR2 mRNA expression was reduced by siRNA for HDAC1 (**Figure 5C**), but not HDAC2 and HDAC3 (**Figure 5D**). As a result, HDAC1 was knocked down by siRNAs in ccRCC cells with FMNL1 overexpression. Results of qRT-PCR and western blot demonstrated that the FMNL1-mediated upregulation of CXCR2 was abolished by HDAC1 silencing (**Figures 5E, F**). The alteration of histone acetylation of CXCR2 promoter was next determined by ChIP. Results showed that histone H3 was acetylated at lysine 9. Downregulation of HDAC1 by siRNAs markedly increased the binding of H3K9ac on the CXCR2 promoter (**Figure 5G**). In clinical samples, high expression of CXCR2 was often observed in ccRCC cases with positive FMNL1 expression (**Figure 5H**). Statistically, a significant positive correlation was found between FMNL1 and CXCR2 expression (**Figure 5H**). In SYSUCC cohort, patients with high CXCR2 expression were likely to live shorter lives, compared to those with low expression of CXCR2 (**Figure 5I**). Furthermore, worst prognosis was identified in ccRCC patients with high expression of FMNL1 and CXCR2 (**Figure 5J**). Collectively, our findings suggest FMNL1 works with HDAC1 to induce the expression of CXCR2 to promote tumor metastasis.

## Discussion

Tumor metastasis is the main cause of cancer-related death. Patients with metastatic ccRCC was accompanied with a significant decrease of survival chance, compared to the ones with localized tumor (14). Although plenty of factors have been identified to be related to ccRCC metastasis (15, 16), few of them were applied in clinical management. Thus, metastatic ccRCC remains one of the challenges to clinicians. More efforts should be paid to unveil the mechanism of ccRCC cell migration. Here in our study, we found that FMNL1 was upregulated by the loss of GATA3 in ccRCC, and function as a prometastatic factor *via* HDAC1-mediated CXCR2 upregulation.

Previous study showed that FMNL1 expression was not restricted to hematopoietic tissues, and overexpression of FMNL1 could be depicted in epithelial cancer cells (5). Using TMA-based IHC in a large cohort of ccRCC patients, we obtained solid evidence that FMNL1 expression was markedly increased in ccRCC tissues, compared with the nontumorous ones. Furthermore, high FMNL1 expression was correlated with poor overall and disease-free survivals. Our data were consistent with previous studies reporting FMNL1 was overexpressed in glioblastoma, lung cancer and nasopharyngeal carcinoma, and was associated with patient prognosis (9–11). According to TCGA data, gene amplification of FMNL1 was rare in ccRCC cells, and is insufficient to support the high expression of FMNL1. Since the mRNA level of FMNL1 was upregulated in ccRCC cells, we determined to investigate the transcriptional regulation of FMNL1 gene in ccRCC. Current literatures reported that FMNL1 was epigenetically modulated by microRNAs, such as miR-16 and miR-143 (9, 17). We performed bioinformatic analyses and found binding sites of GATA3 in the FMNL1 promoter. *In vitro* experiments showed that ectopic expression of GATA3 dramatically suppressed the expression of FMNL1. A reverse correlation was confirmed between GATA3 and FMNL1 expressions in clinical samples. Gene silencing of GATA3 was documented in several cancers (18–20) and re-expression of GATA3 was capable of inhibiting tumor metastasis (21–23). However, the precise mechanism of FMNL1 regulation by GATA3 was not clear and requires further investigation.

*In vitro* and *in vivo* data demonstrated that FMNL1 overexpression was able to promote cell migration and tumor metastasis, which was in line with previous studies showing that RNAi-mediated silencing of FMNL1 markedly weakened the cell migration potent in human cancer cells (6, 9, 10). The role of FMNL1 in maintenance of Golgi structural integrity indicated that FMNL1 exerts biological functions might be thought protein interaction to affect the stabilization and degradation of certain partner. Protein interactome studies revealed several candidates that FMNL1 may interact with (24). However, such protein interaction did not affect the posttranslational modulation of target proteins. Interestingly, in nasopharyngeal carcinoma cells, FMNL1 prevented the nuclear translocation of HDAC1 through binding to profilin2 to subsequently relieve the HDAC1-induced translational suppression of MTA1 (9). In the present study, FMNL1 enhanced cell migration and invasion *via* upregulation of CXCR2. Further investigation revealed that HDAC1 was required for the oncogenic function of FMNL1. But how FMNL1 interacts with HDAC1 to increase the transcript of CXCR2 in ccRCC cells is still obscure. Previous studies showed that CXCR2 expression was negatively associated with the prognosis of ccRCC patients (25). CXCR2 was involved in the tumor growth and stemness maintenance in ccRCC (26), and chemoresistance, tumor metastasis and angiogenesis in other cancers (27, 28). Most of the studies focus on the prometastatic activity of FMNL1 demonstrated that enforced expression of FMNL1 triggered the EMT process. Unsurprisingly, our data showed that FMNL1 increased the expression of mesenchymal markers but decreased the expression of epithelial markers. These data suggest FMNL1-mediated tumor metastasis may be achieved through similar mechanism and could be developed into a potential therapeutic target.

To date, investigations on the role of FMNL1 in solid cancers, such as ccRCC, lung cancer and nasopharyngeal carcinoma, indicate FMNL1 as a promising prognostic marker to predict the outcomes of cancer patients. Our clinical data revealed that increased expression of CXCR2, one of the downstream effectors of FMNL1, was reversely correlated with the overall survival of ccRCC patients. Combination of high FMNL1 and high CXCR2 may help to distinguish ccRCC patients with worse prognosis. Collectively, our data suggest FMNL1 serve as a promising prognostic factor in ccRCC and function as an oncogene to promote tumor metastasis through upregulation of CXCR2. Targeting FMNL1/HDAC1/CXCR2 axis may represent a potential therapeutic strategy in the clinical management of ccRCC.

## Data Availability Statement

The original contributions presented in the study are included in the article/**Supplementary Material**. Further inquiries can be directed to the corresponding authors.

## Ethics Statement

The studies involving human participants were reviewed and approved by Sun Yat-sen University Cancer Center Institute Research Ethics Committee. The patients/participants provided their written informed consent to participate in this study. All animal studies were approved by the Medical Experimental Animal Care Commission of Sun Yat-sen University Cancer Center.

## Author Contributions

CZ and YC conceived and designed the experiments. M-FZ, Q-LL, Y-FY, and YC performed the experiments. Y-FY, YC, and CZ analyzed the data. M-FZ and CZ wrote and revised the manuscript. All authors contributed to the article and approved the submitted version.

## Funding

This study was supported by the Medical Scientific Research Foundation of Guangdong Province of China (B2018243).

## Conflict of Interest

The authors declare that the research was conducted in the absence of any commercial or financial relationships that could be construed as a potential conflict of interest.
