# Supplementary material for: FMNL1 Exhibits Pro-Metastatic Activity via CXCR2 in Clear Cell Renal Cell Carcinoma
Source: Front Oncol. 2020 Nov 26;10:564614. doi: 10.3389/fonc.2020.564614 (PMC7726248; doi:10.3389/fonc.2020.564614)
Supplement: Supplementary file 1 [file DataSheet_1.pdf]

Supplementary table 1. Association between FMNL1 expression and clinicopathological features of ccRCC patients in SYSUCC cohort.

Variable	Cases	FMNL1		*P* value
		Low expression	High expression	
Fuhrman				**0.013**
I	46	27 (58.7%)	19 (41.3%)	
II	182	83 (45.6%)	99 (54.4%)	
III	59	20 (33.9%)	39 (66.1%)	
IV	19	4 (21.1%)	15 (78.9%)	
Sarcomatoid differentiation				0.240
No	292	130 (44.5%)	162 (55.5%)	
Yes	14	4 (28.6%)	10 (71.4%)	
Necrosis				0.272
No	228	104 (45.6%)	124 (54.4%)	
Yes	78	30 (38.5%)	48 (61.5%)	
Lymph node invasion				**0.008**
No	283	130 (45.9%)	153 (54.1%)	
Yes	23	4 (17.4%)	19 (82.6%)	
Vascular invasion				0.070
No	280	127 (45.4%)	153 (54.6%)	
Yes	26	7 (26.9%)	19 (73.1%)	
T stage				0.184
I	205	95 (46.3%)	110 (53.7%)	
II	64	29 (45.3%)	35 (54.7%)	
III	29	8 (27.6%)	21 (72.4%)	
IV	8	2 (25.0%)	6 (75.0%)	

Supplementary figures

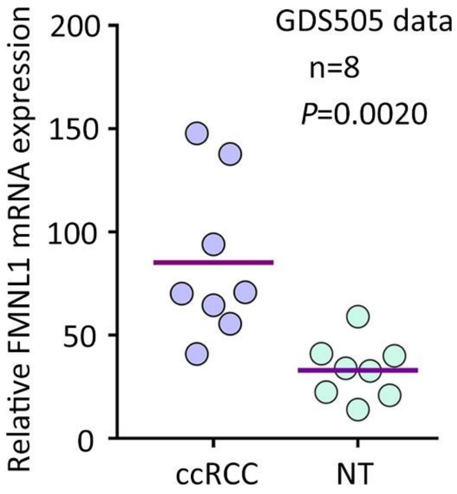

Supplementary figure 1. The expression of FMNL1 mRNA in public database (GDS505). A total of eight paired ccRCC tissues and corresponding nontumorous tissues were subjected to RNA sequencing. The relative expression of FMNL1 was indicated.

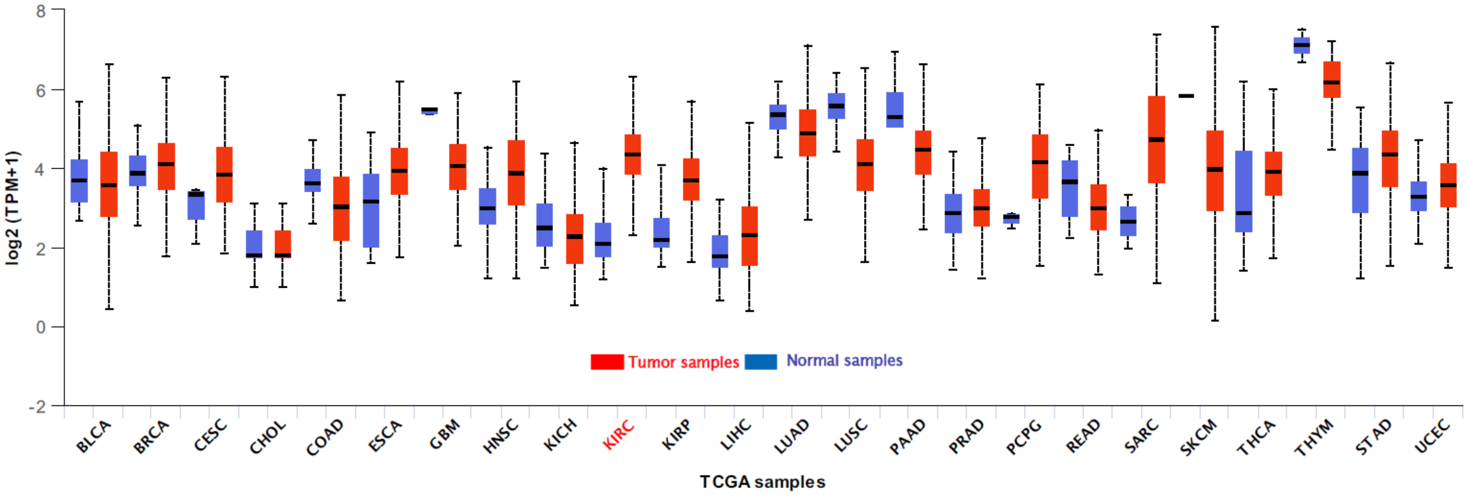

Supplementary figure 2. The expression of FMNL1 mRNA in TCGA cancers. The expression of FMNL1 mRNA in tumor tissues and nontumorous tissues was shown by Box plots.

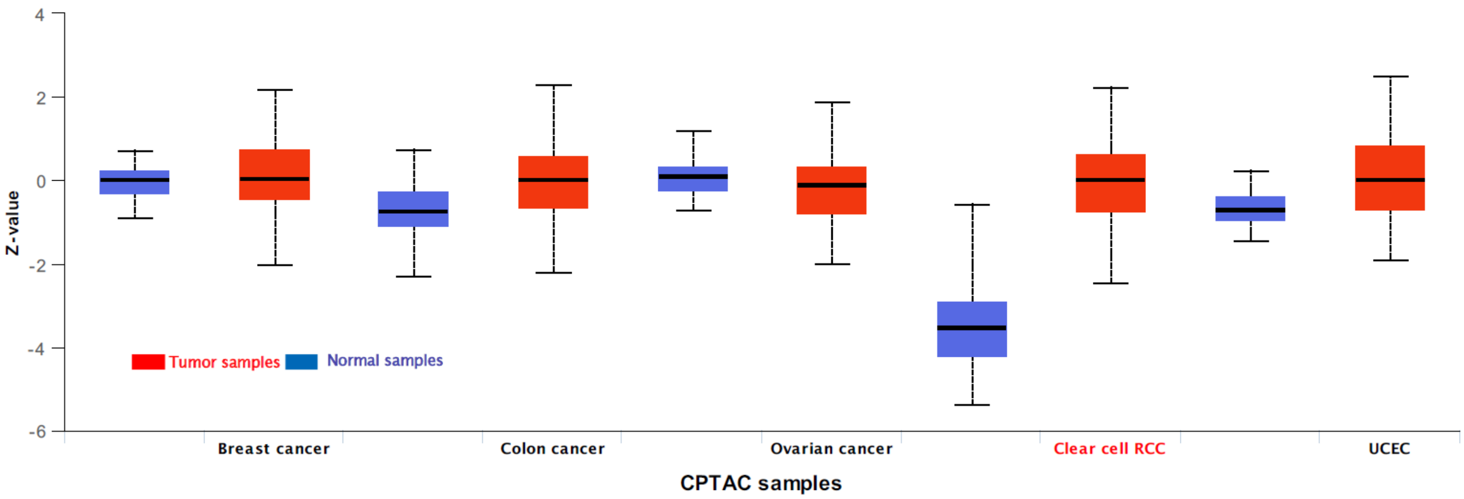

Supplementary figure 3. The expression of FMNL1 protein in Clinical Proteomic Tumor Analysis Consortium (CPTAC) database. The expression of FMNL1 promtein in tumor tissues and nontumorous tissues was shown by Box plots.

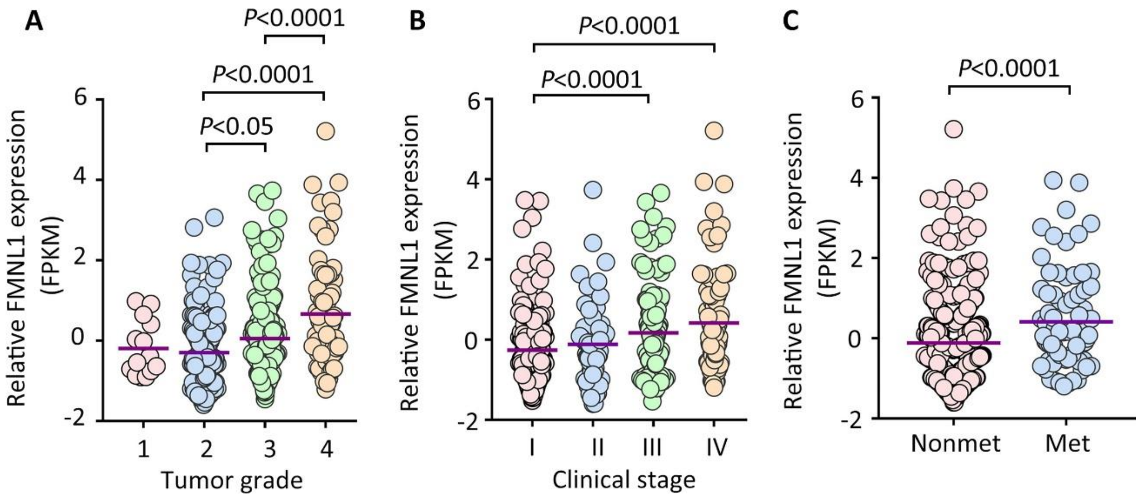

Supplementary figure 4. The correlation between FMNL1 expression and tumor stage, tumor grade and tumor metastasis of ccRCC patients in TCGA cohort. Patients were divided into groups according to tumor grades, clinical stages, and metastatic states. The expression of FMNL1 mRNA was indicated and compared.

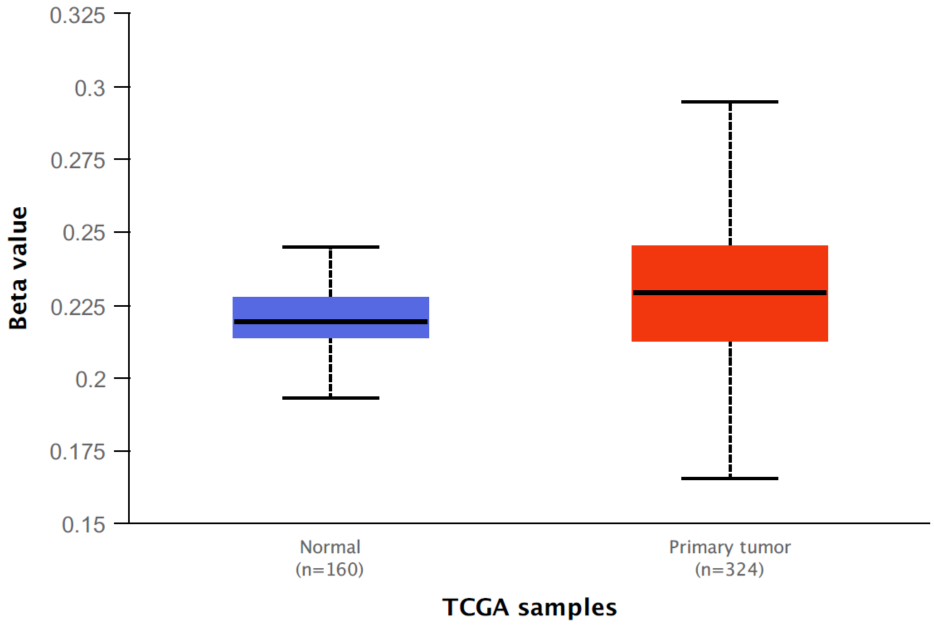

Supplementary figure 5. The status of promoter methylation of FMNL1 in TCGA cohort. The promoter methylation levels of FMNL1 in kidney cancer tissues and normal tissues were shown.

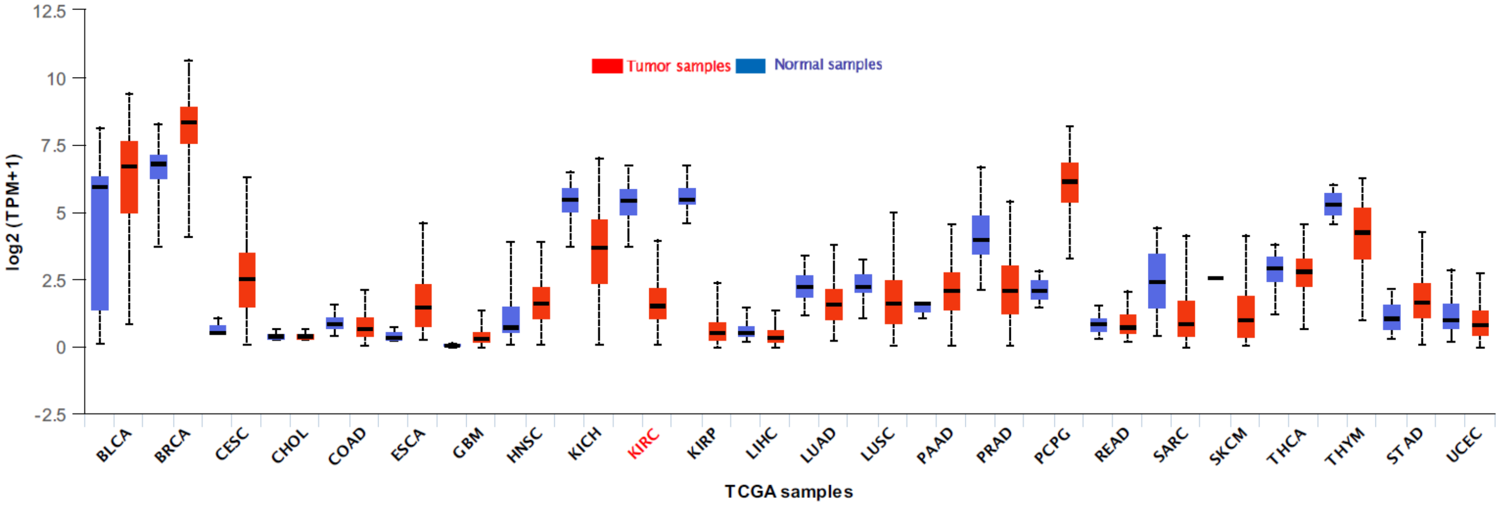

Supplementary figure 6. The expression of GATA3 mRNA in TCGA cancers. The expression of GATA3 mRNA in tumor tissues and nontumorous tissues was shown by Box plots.

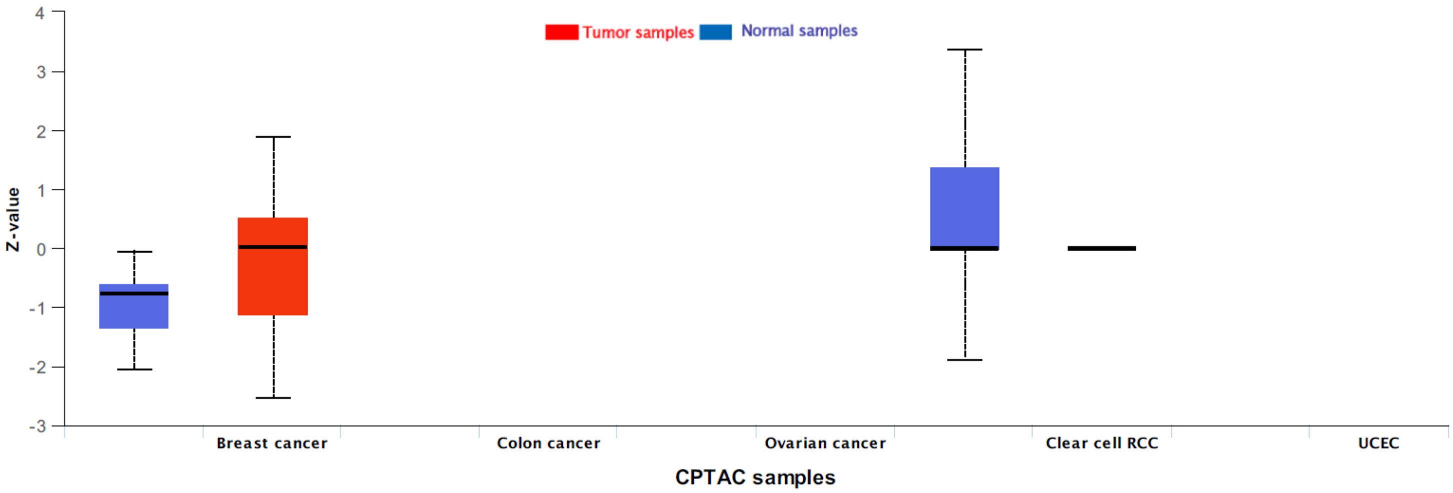

Supplementary figure 7. The expression of GATA3 protein in Clinical Proteomic Tumor Analysis Consortium (CPTAC) database.
